# Effects of Dark Septate Endophytes on the Performance and Soil Microbia of *Lycium ruthenicum* Under Drought Stress

**DOI:** 10.3389/fpls.2022.898378

**Published:** 2022-06-02

**Authors:** Chao He, Tingting Han, Ling Tan, Xianen Li

**Affiliations:** ^1^Institute of Medicinal Plant Development, Chinese Academy of Medical Sciences and Peking Union Medical College, Beijing, China; ^2^Xiyuan Hospital, Chinese Academy of Traditional Chinese Medicine, Beijing, China

**Keywords:** dark septate endophytes, plant performance, soil microbial community composition, drought, *Lycium ruthenicum*, symbiosis

## Abstract

In the current study, we explored the effects of dark septate endophytes (DSE) (*Neocamarosporium phragmitis, Alternaria chlamydospore*, and *Microascus alveolaris*) on the performance and rhizosphere soil microbial composition of *Lycium ruthenicum* Murr under drought stress. Differences in plant growth and physiological indexes, soil parameters, and microbial composition under different treatments were studied. Three DSE species could form good symbiotic relationships with *L. ruthenicum* plants, and the symbionts depended on DSE species and water availability. Inoculation of DSE had the greatest benefit on host plants under drought conditions. In particular, *N. phragmitis* and *A. chlamydospore* had a significant positive influence on the biomass, morphological and physiological indexes of host plants. Additionally, the content of arbuscular mycorrhiza (AM) fungi, gram-negative bacteria, and actinomycetes in the soil was significantly elevated after DSE inoculation in the absence of water. Based on a variance decomposition analysis, DSE was the most important factor affecting the growth and physiological parameters of host plants, and DSE inoculation combined with water conditions significantly affected the contents of soil microbial communities. Structural equation model (SEM) analysis showed that the positive effects of DSE on *L. ruthenicum* varied with DSE species and plant parameters under different water conditions. These results are helpful to understand the ecological function of DSE and its potential application in the cultivation of *L. ruthenicum* plants in drylands.

## Introduction

The environment is the substrate for plants to survive. Appropriate environmental conditions can ensure or promote plant growth and development, while adverse environmental conditions can inhibit plant growth and even lead to plant death (Cherif et al., 2015). Plants have both direct and indirect responses to environmental changes. In recent years, the symbiotic relationship between endophytic fungi and host plants has attracted much attention as an indirect way to mitigate the adverse effects of environmental change on plants. The plant provides the nutrition for the growth of the endophytic fungi, which can adjust the hormone level of the host plant, modify the plant growth, and improve the ability of the host plant to withstand harsh environments (Azcón-Aguilar et al., 2003; Kivlin et al., 2013; Shi et al., 2015). Hence, the selection of fungal strains suitable for plant growth can effectively promote plant performance and ecological adaptability (Berthelot et al., 2017).

Dark septate endophytes (DSE) belong to the *Ascomycetes* and commonly colonize the roots of both mycorrhizal and non-mycorrhizal plants (Jumpponen and Trappe, 1998; Addy et al., 2005). These endophytes are characterized by melanized septate hyphae and microsclerotia (Mandyam et al., 2010; Muthukumar and Sathya, 2017). Studies have shown that most plants can form complex and changeable symbiotic relationships with fungi, and symbiotic fungi help to enhance the adaptability of plants to drought stress (Berthelot et al., 2017). Wu et al. (2010) had found that inoculated DSE could promote plant growth and medicinal ingredients contents of *Saussurea involucrata*. Zhang et al. (2012) had reported that inoculated LBF-2 strain promoted the branch number, leaf number, and chlorophyll content and enhanced the drought resistance of *Lycium ruthenicum*. Yakti et al. (2018) had found that DSE inoculation promoted the uptake of organic nitrogen sources and increased tomato biomass. Li et al. (2018) had inoculated 9 DSE species into *Ammopiptanthus mongolicus*, and only 3 species had positive effects on plant growth, which reduced the damage of drought stress to plants, while the other DSE showed neutral or negative effects. Zuo et al. (2020) had found that the growth-promoting effect of DSE on *Hedysarum scoparium* significantly varied based on DSE species and inoculation dose. Newsham (2011) had considered that the inoculation effect of DSE was affected by DSE strains, host plant species, experimental conditions, and other factors, but the specific mechanism was unclear. Additionally, studies have shown that inoculation with endophytic fungi can affect the microbial composition and distribution in rhizosphere soil, while rhizosphere soil microorganisms have the functions of decomposing soil organic matter and regulating soil nutrients, thereby assisting host plants in adapting to different adverse environments (Bai et al., 2015). Albertsen et al. (2006) found that *Glomus intraradices* positively influenced the bacterial and saprotrophic fungal biomass in a root-free sand environment. Inoculation with arbuscular mycorrhizal fungi could change the composition of root exudates and alleviate the effects of diseases related to continuous cropping (Lu and Wu, 2019). Although the positive effects of inoculation with beneficial fungi on plant growth and rhizosphere-associated microorganisms have been widely reported (Trabelsi and Mhamdi, 2013; He et al., 2019a), little is known about the effects of DSE on rhizosphere microbial communities.

*Lycium ruthenicum* (Solanaceae) is a perennial spiny shrub widely distributed in arid areas of northwest China. It is a natural medicinal desert plant with high nutritional and medicinal value (Zheng et al., 2010). Drought stress often adversely affects organisms, causing reactive oxygen damage (Bartels and Sunkar, 2005). Among other mechanisms, superoxide dismutase (SOD) is an important protective enzyme (Khan et al., 2018; Saleem et al., 2018). Some studies have found that DSE can improve plant resistance by regulating SOD activity, glutathione, soluble protein, and cell wall structure and produce a large amount of melanin under stress (Sharma et al., 2012; Zhao et al., 2015). Here, we hypothesize that inoculated DSE can either enhance the plant growth or modify the soil microbial community of *L. ruthenicum*, and DSE may be more positive effects under water deficiency than under normal water. Hence, we studied the effects of DSE inoculation under different water conditions on plant biomass and morphological characteristics, physiological characteristics, soil properties, and soil microbial community. We expect our results to reveal (1) Can DSE alleviate the damage caused by water shortage to *L. ruthenicum* plants? (2) What is the relationship between DSE, *L. ruthenicum* and soil microorganisms under different water conditions?

## Materials and Methods

### Biological Materials and Growth Substrate

Three fungi were isolated from the roots of *L. ruthenicum* growing in the desert environment of northwest China. Their ITS sequences have been uploaded to GenBank with accession numbers MW548084 for *Neocamarosporium phragmitis* (NP), MW548074 for *Alternaria chlamydospora* (AC), and MW548081 for *Microascus alveolaris* (MA) (Han et al., 2021). These fungi are deposited in the culture collection of the Institute of Medicinal Plant Development, Chinese Academy of Medical Sciences, Beijing, China. Mature seeds of *L. ruthenicum* were collected from Gansu Province, China, and stored at 4°C.

The growth medium used was a mixture of 1:2 (W:W) sand (<2 mm) and soil, which was the original soil for the natural growth of *L. ruthenicum* in north China. Physicochemical properties of the growth medium: organic matter 20.23 mg/g, available nitrogen 87.54 mg/kg, and available phosphorus 8.11 mg/kg, respectively.

### Experimental Design

The experiment was a two-factor, completely randomized block design (four inoculation treatments × two water treatments) with five replications for each treatment. The inoculation treatments included *N. phragmitis, A. chlamydospora, M. alveolaris*, and control without inoculation. Water treatments were normal water (WW) and drought stress (DS). A total of 40 pots were used.

Mature seeds of *L. ruthenicum* were soaked in 75% ethanol for 2 min, and soaked in 2.5% sodium hypochlorite for 8 min with constant stirring. Sterilized seeds were rinsed with sterile water and cultured in Agar medium (containing 10 g/L Agar) at 25°C. After the seeds germinated, the seedlings were transplanted into plastic pots (18 cm in height and 15 cm diameter, 3 seedlings for each pot) with 700 g of the non-sterile medium.

The fungal inoculum was prepared by aseptically culturing DSE isolates using a potato dextrose agar (PDA) medium. For inoculation pots, two 4-mm patches were cut along the edge of the active colonies on the culture medium and inoculated within 1 cm of the roots of seedlings. The control pots were inoculated with patches excised from the medium without fungus. Specifically, each plastic pot was first added with 500 g soil and topped with two 4-mm fungal cakes, and then covered with 200 g soil. All inoculations were proceeded on a clean bench, and all potted plants were placed in an incubator with a photoperiod of 14/10 h, 27/22°C (day/night), light intensity of 2500 lx and an average relative humidity of 55%. The experiment was arranged from April 12, 2021 to harvest on September 12, 2021, for a period of 5 months.

After 1 month, all test seedlings were divided into two groups, half of which (control and inoculation treatment) kept 60% field water capacity (WW), and the other half kept 30% field water capacity (DS). During the experiment, water loss was replenished daily with sterile water, and soil moisture was maintained by periodic weighing.

### Plant Biomass and Morphological Index

The morphological index such as plant height, number of leaves and spines of each replicate were observed at harvest time. Chlorophyll content of the top fourth leaves per plant was determined by SPAD-502 Chl meter (Konica Minolta Sensing, Osaka, Japan). The shoots and roots of per plant were harvested separately and rinsed with tap water. Individual root samples were floated in a plexiglass tray about 1 cm deep, and scanned with a desktop scanner. Total root length and mean diameter were measured by a Win-RHIZO image analysis system. Root and shoot samples were weighed after being kept in an oven at 65°C for 48 h, and the total biomass was calculated.

### Plant Physiological Characteristics

After the plant growth parameters were tested, the fresh leaves of the different treatments were placed in marked sterile self-sealing bags (5 g of fresh leaves were collected for each treatment) and stored in a 4°C refrigerator in preparation for testing the active ingredients of the plants.

Glutathione (GSH) content was determined using the dithiobinitrobenzoic acid method (Anderson, 1985). The 0.5 g of fresh leaves were ground with 5% sulfosalicylic acid at 4°C, and then the crude extract was centrifuged at 10,000 × *g* for 10 min, and the optical density of the supernatant was determined at 421 nm. The GSH content was calculated based on a standard curve.

Superoxide dismutase (SOD) activity was determined *via* photochemical reduction with nitroblue tetrazolium (Harrach et al., 2008). About 0.5 g of fresh leaves was homogenized in 5 ml of 50 mM KH_2_PO_4_ buffer (pH 7.8), which contained frozen EDTA (0.2 mM) and polyvinylpyrrolidone (2%) and ground with a pre-cooled abrasive tool. The supernatant was obtained for enzymatic assays after centrifugation of the homogenate for 30 min at 15,000 × *g* using a centrifuge. Take 3 ml of the above enzyme reaction system solution under dark light and transfer it into a test tube, which is placed in a reaction cell with tin foil on the wall of the reaction cell, and each test tube should be placed in a position where it receives the same light intensity after illumination. Add 25–30 μl of enzyme solution to each tube. Illuminate at 25–30°C with a fluorescent tube of light intensity 4,000 l × (15 W fluorescent lamp can be used), and after 15–20 min, the color change appears. Stop the light illumination. Measure the transmittance colorimetrically at 560 nm wavelength.

Soluble protein content was determined *via* Coomassie brilliant blue method (Zhang et al., 2015). The 0.5 g of fresh leaves was ground using quartz sand, and together with 5 ml of 50 mM phosphate buffer (pH 7.0), and the homogenate was centrifuged at 4,000 × *g* for 10 min (4°C). The absorbance of the supernatant was determined at 595 nm *via* a spectrophotometer.

Malondialdehyde (MDA) content was measured using the thiobarbituric acid (TBA) method (Peever and Higgins, 1989). About 0.5 g of fresh leaves was homogenized and centrifuged for 10 min at 12,000 × *g*, and then placed in 5 ml trichloroacetic acids (10 %). The mixture containing 2 ml of supernatant and 2 ml of TBA (0.5 %) was put into boiling water for 15 min, then quickly cooled and centrifuged at 12,000 × *g* for 10 min. The absorbance of the supernatant was measured at 450, 532, and 600 nm *via* a spectrometer. The content of MDA was calculated using the following equation:


$$\begin{array}{l} C\left(\mu mmol/L\right)=6:45\left(OD_{532}-OD_{600}\right)-0.56OD_{450} \end{array}$$

Proline content was determined with reference to the method given by Bates et al. (1973). About 0.5 g of fresh leaves was homogenized with 3% sulfosalicylic acid solution (5 mL), and the homogenate was heated in a boiling water bath for 10 min and filtered through filter paper. About 2 ml of the filtrate was heated at 100°C for 30 min with 2 ml of glacial acetic acid and 2 ml of acid indene, and the reaction was ended in an ice bath. The reaction mixture was extracted with toluene (4 ml) and centrifuged at 3,000 × *g* for 5 min. The extract was then measured for absorbance (at 520 nm) with a spectrophotometer.

### Indole-3-Acetic Acid Content

Fresh roots (about 100 mg) were treated with 1 ml 0.01 mol/L pre-chilled phosphate buffer (pH 7.2). Centrifuge the homogenate at 3,000 × g for 20 min at 4°C using a centrifuge and collect the supernatant. The IAA content was measured with an IAA ELISA kit (Mlbio, Shanghai, China). Absorbance was determined at 450 nm *via* an Epoch 2 microplate reader (BioTek, Winooski, USA).

### DSE Root Colonization

DSE colonization was determined according to the Phillips and Hayman method (1970). Briefly, fresh roots were washed in tap water and cut into 0.5-cm long segments. For each sample, 10 randomly selected root segments were examined to confirm that the roots were colonized by the respective fungal inocula. Root segments was treated with 10% (w/v) KOH solution (100°C, 1 h), and stained with 0.5% (w/v) acid fuchsine (90°C, 20 min). Fifty root segments per sample were sequentially observed under 20 × and 40 × magnification microscopes.

### Soil Physicochemical Properties

Soil pH was measured with a precision acidometer (PHS-3C). The soil organic matter content was assessed by the combustion method, and the soil samples were placed in a muffle furnace (TMF-4-10T) at 550°C for 4 h (Heiri et al., 2001). The content of available nitrogen and available phosphorus were determined using the alkaline hydrolysis-diffusion method and chlorostannous-reduced molybdophosphoric blue method, respectively (Bever et al., 1996).

### Composition of Soil Microbial Community

The analysis of soil phospholipid fatty acids (PLFAs) composition was used to assess rhizosphere microbial community structure. Specifically, lipids were extracted from frozen soil samples weighing ~8.0 g using a modified (Bossio and Scow, 1998) method using 23 ml of chloroform-methanol-phosphate buffer at a volume ratio of 1:2:0.8. The extracts were separated on a 3 ml silica column (0.5 g silicic acid) using chloroform (5 m), acetone (20 ml), and methanol (5 ml). The methanol solution was collected and released in the presence of nitrogen. Phospholipids were sequentially saponified and methylated to form fatty acid methyl esters (FAMEs). Individual FAMEs were identified and quantified *via* gas chromatography (GC, America, Agilent 6890N) equipped with MIDI software package Sherlock MIS Version 4.5 (MIDI Inc., Newark, Delaware, USA) and were analyzed for PLFAs (He et al., 2019b).

### Data Analysis

For the current experiment, A two-way analysis of variance (ANOVA) was used to assess the effects of DSE, water regime, and their interaction on plant biomass, morphological index, physiological index, soil physicochemical properties, and microbial community. The data shown in the figure is an average of at least five repetitions. SPSS 21.0 software was used for all of the above analyses. The effects of each factor on the growth and physiological index of *L*. *ruthenicum* were estimated by variation partitioning analysis (VPA). Mantel test and structural equation modeling (SEM) were performed to test the effects of DSE species, soil physicochemical parameters, and microorganisms, on the growth and physiological parameters of *L*. *ruthenicum* in R-3.2.2 package ecodist (Goslee and Urban, 2007) and AMOS 21.0 (maximum likelihood).

## Results

### DSE Root Colonization

**Figure 1:**
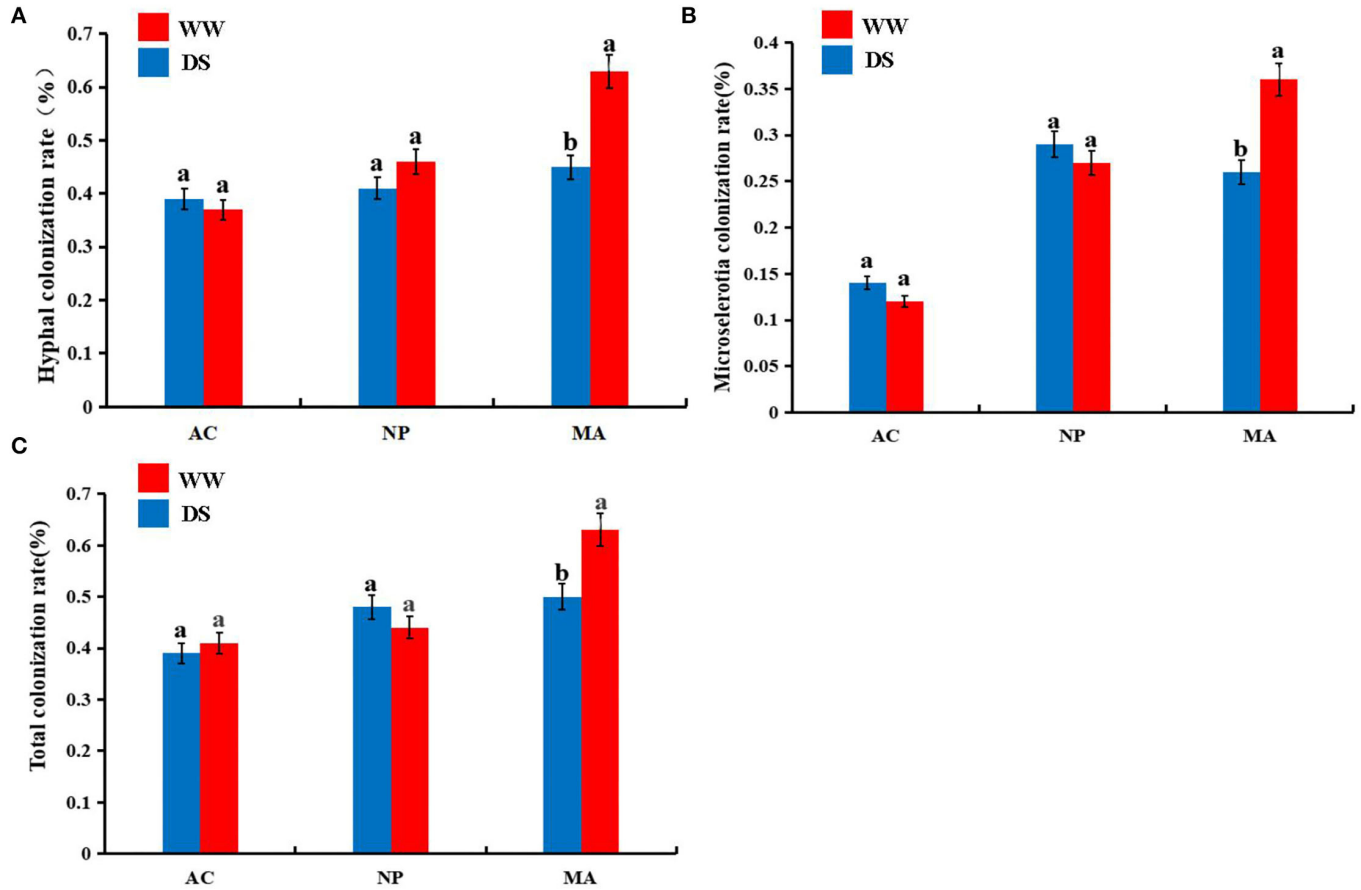
Colonization rates of dark septate endophytes (DSE) in the roots of *Lycium ruthenicum*. **(A)** hyphal colonization; **(B)** microsclerotial colonization; **(C)** total colonization. AC, *Alternaria chlamydospore*; NP, *Neocamarosporium phragmitis*; MA, *Microascus alveolaris*; WW, well-watered; DS, drought stress, respectively. The a, b letters indicate significant difference at P <0.05 by Duncan's multiple-range tests.

Five months after inoculation with DSE strain, the roots of *L*. *ruthenicum* plants were observed. No DSE colonization structure was found in the control plants, while DSE colonization structure could be formed in the root cortex and vascular tissue of the host plants inoculated with DSE (Supplementary Figure 1). Two-way ANOVA results showed that DSE significantly affected hyphal colonization and microsclerotia colonization rate. However, water conditions and the interaction between DSE and water were not significantly correlated with DSE colonization parameters (Table 1). There were no significant differences in hyphal, microsclerotia, and total colonization between the two water regimes after AC or NP inoculation. However, the hyphal, microsclerotia, and total colonization were significantly higher after MA inoculation under normal water than under drought stress (Figure 1).

**Table 1:** Two-way ANOVA of the effect of DSE and water condition on DSE colonization rate of *Lycium ruthenicum*.

	**DSE**		**Water**		DSE*Water	
	***F***	***P***	***F***	***P***	***F***	***P***
Hyphal colonization rate (%)	5.37	*	3.01	NS	4.28	NS
Microselerotia colonization rate (%)	5.26	*	3.28	NS	3.92	NS
Total colonization rate (%)	4.88	NS	2.64	NS	3.41	NS

The * symbol indicates the significant difference P <0.05.

### Plant Biomass and Morphological Parameters

**Figure 2:**
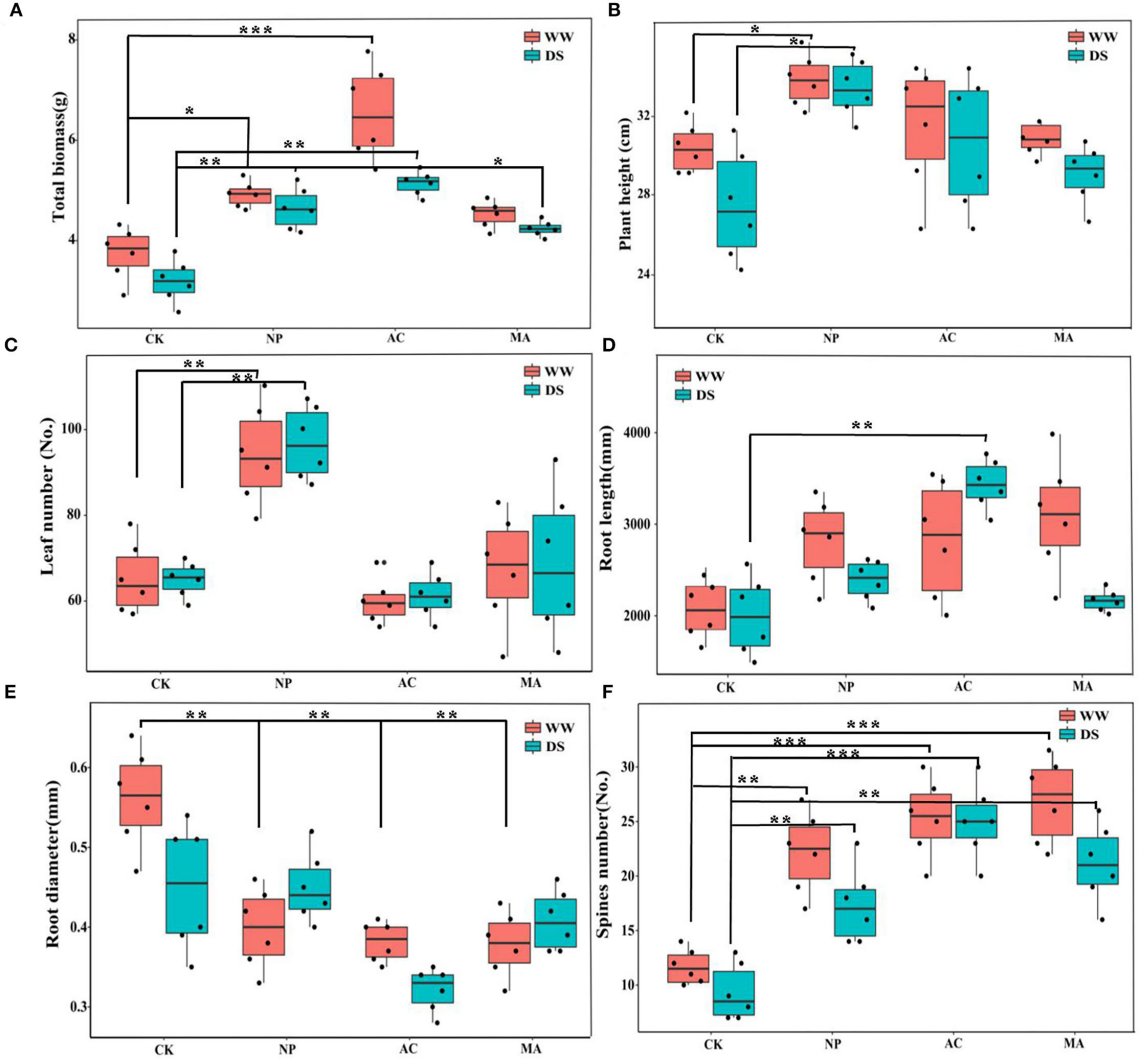
Effect of different dark septate endophytes (DSE) on morphological parameters of growth of *Lycium ruthenicum* seedlings. CK, non-inoculated plants; NP, plants inoculated with *Neocamarosporium phragmitis*; AC, plants inoculated with *Alternaria chlamydospore*; MA, plants inoculated with *Microascus alveolaris*; WW, well-watered; DS, drought stress, respectively. The total biomass of *Lycium ruthenicum* seedlings **(A)**, Plant height of *Lycium ruthenicum* seedlings **(B)**, Leaf number of *Lycium ruthenicum* seedlings **(C)**, Root length of *Lycium ruthenicum* seedlings **(D)**, Root diameter of *Lycium ruthenicum* seedlings **(E)**, and Spines number of *Lycium ruthenicum* seedlings **(F)**. The * symbol indicates the significant difference *P* < 0.05. The ** symbol indicates the significance level intervenes between *P* < 0.01. The *** symbol indicates difference is very significant, *P* < 0.001.

The growth parameters of *L. ruthenicum* seedlings were significantly affected by DSE, and the total biomass and root length were significantly related to water, while the total biomass, root length, and number of spines were affected by the interaction between DSE and water (Table 2).The biomasses of AC and NP inoculated under WW were higher than that of other treatments, while the biomass of plants inoculated with DSE under DS condition was significantly higher than that of control plants (Figure 2A). Compared with the control plants, plant height was increased after inoculation with NP, but it did not change after inoculation with AC and NP (Figure 2B). Leaf number increased after inoculation with NP, but it did not change after inoculation with AC and MA compared with the control plants (Figure 2C). Under WW, there was no difference in the root length among all treatments, while under DS, the root length inoculated with AC was higher than that of other treatments (Figure 2D). Root diameter inoculated with DSE decreased under WW compared with the control plants. Under DS, only AC inoculation decreased root diameter (Figure 2E). The number of spines inoculated with DSE was higher than that of the control plants, regardless of the water regime (Figure 2F).

**Table 2:** Two-way ANOVA of the effect of DSE and water condition on plant growth parameters of *Lycium ruthenicum*.

	**DSE**		**Water**		DSE*Water	
	***F***	***P***	***F***	***P***	***F***	***P***
Total biomass (g)	7.89	**	5.19	*	5.16	*
Plant height (cm)	5.42	*	2.13	NS	0.89	NS
Leaf number (No.)	6.02	*	2.78	NS	3.71	NS
Root length (mm)	5.23	*	5.41	*	5.42	*
Root diameter (mm)	5.67	*	4.38	NS	3.05	NS
Spines number (No.)	6.87	*	3.36	NS	5.17	*

The * symbol indicates the significant difference P <0.05. The ** symbol indicates the significance level intervenes between P <0.01.

### Physiological Parameters

**Figure 3:**
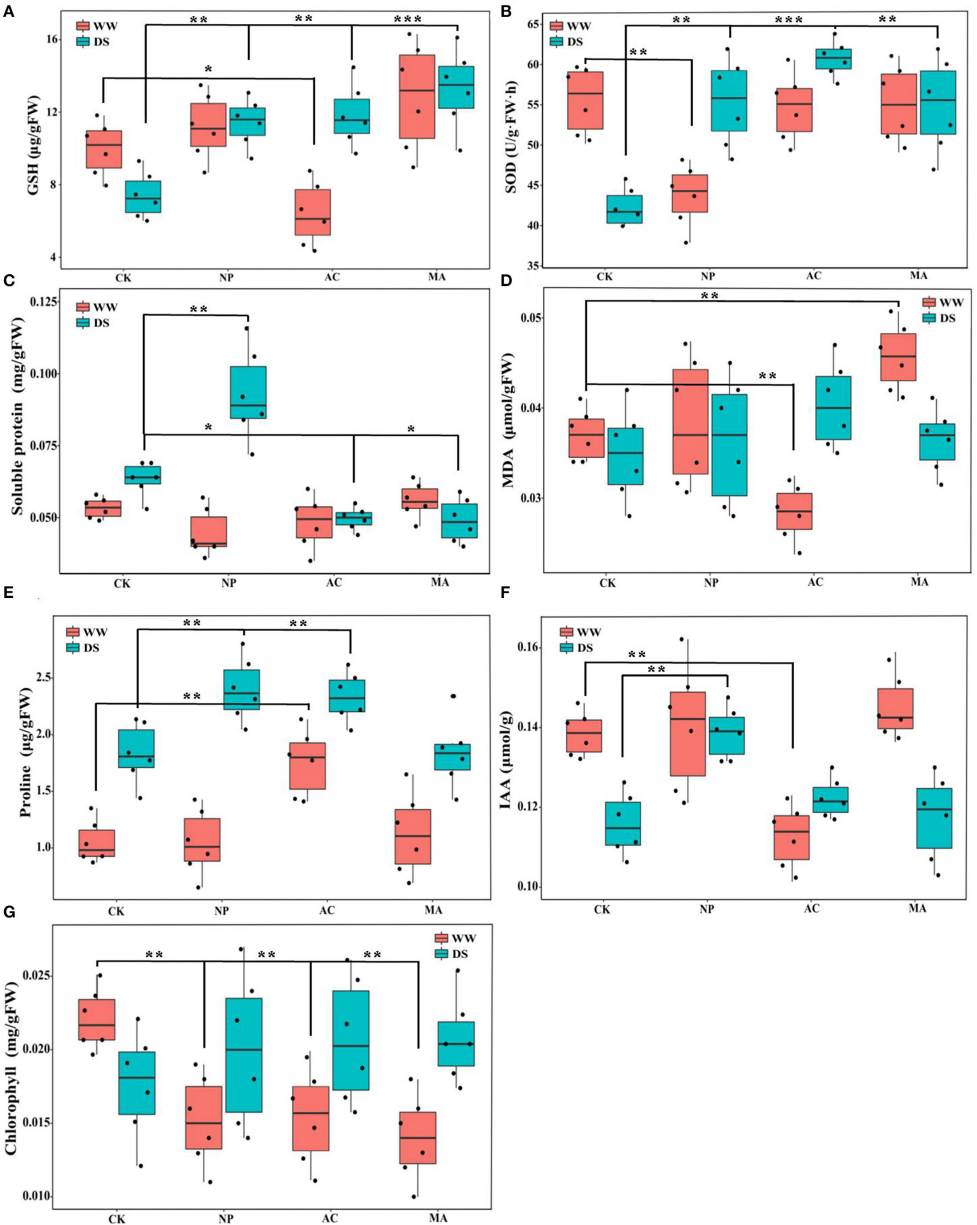
Effect of different dark septate endophytes (DSE) on physiological active ingredient parameters in *Lycium ruthenicum* seedlings. CK, non-inoculated plants; NP, plants inoculated with *Neocamarosporium phragmitis*; AC, plants inoculated with *Alternaria chlamydospore*; MA, plants inoculated with *Microascus alveolaris*; WW, well-watered; DS, drought stress, respectively. GSH content of *Lycium ruthenicum* seedlings **(A)**, SOD content of *Lycium ruthenicum* seedlings **(B)**, Soluble protein content of *Lycium ruthenicum* seedlings **(C)**, MDA content of *Lycium ruthenicum* seedlings **(D)**, Proline content of *Lycium ruthenicum* seedlings **(E)**, IAA content of *Lycium ruthenicum* seedlings **(F)**, and Chlorophyll content of *Lycium ruthenicum* seedlings **(G)**. The * symbol indicates the significant difference *P* < 0.05. The ** symbol indicates the significance level intervenes between *P* < 0.01. The *** symbol indicates difference is very significant, *P* < 0.001.

Two-way ANOVA showed that DSE significantly affected the contents of GSH, SOD, MDA, proline, and chlorophyll, while water conditions significantly affected the contents of GSH, soluble protein, proline, IAA, and chlorophyll. However, the interaction between DSE and water had no significant effect on the active ingredient parameters of *L. ruthenicum* seedlings (Table 3). Under WW, the GSH content with AC inoculation was lower than that of the control seedlings, and the GSH content with DSE inoculation was higher than that of the control plants under DS (Figure 3A). Under WW, for NP inoculation, the activity of SOD was lower than that of other treatments, while the activity of SOD with DSE inoculation was higher than that of the control plants under DS (Figure 3B). In terms of soluble protein content, there was no difference between all treatments under WW, while the soluble protein content with NP inoculation was higher than that of other treatments, and the soluble protein content with AC and MA inoculation was lower than that of the control plants under DS (Figure 3C). Under WW, the MDA content with AC inoculation was lower than that of the control plants, and the MDA content with MA inoculation was higher than that of the control plants, while there was no difference in MDA contents among all treatments under DS (Figure 3D). The proline content with AC inoculation was higher than that of other treatments, and the proline content with NP and AC inoculation was higher than that of the control plants under WW (Figure 3E). Under WW, the IAA content with AC inoculation was lower than that in other treatments, while the IAA content with NP inoculation was higher than that in other treatments under DS (Figure 3F). Under WW, the chlorophyll content with DSE inoculation was higher than that of the control plants, while there was no difference in chlorophyll contents among all treatments under DS (Figure 3G).

**Table 3:** Two-way ANOVA of the effect of DSE and water condition on plant physiological parameters of *Lycium ruthenicum*.

	**DSE**		**Water**		DSE*Water	
	***F***	***P***	***F***	***P***	***F***	***P***
GSH (μg/gFW)	8.05	**	5.23	*	4.62	NS
SOD (U/g · FW · h)	5.12	*	4.08	NS	3.41	NS
Soluble protein (mg/gFW)	3.89	NS	5.05	*	1.25	NS
MDA (μmol/gFW)	6.48	*	3.82	NS	2.89	NS
Proline (μg/gFW)	5.29	*	9.26	**	4.66	NS
IAA (μmol/g)	4.33	NS	5.37	*	0.26	NS
Chlorophyll (mg/gFW)	8.60	**	6.22	*	4.03	NS

The * symbol indicates the significant difference P <0.05. The ** symbol indicates the significance level intervenes between P <0.01.

### Soil Physicochemical Parameters

**Figure 4:**
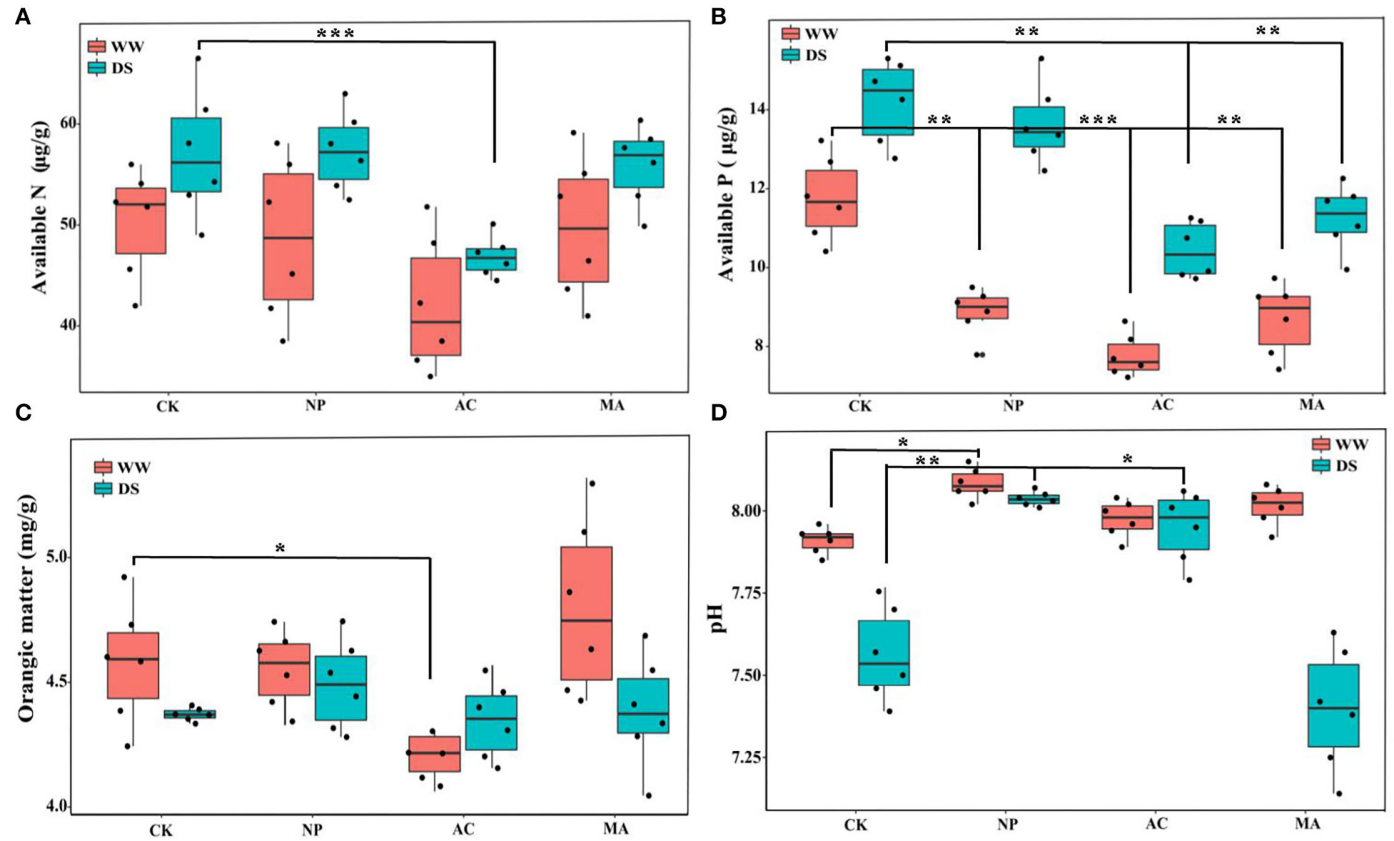
Effects of different dark septate endophyte (DSE) on soil physicochemical parameters of *Lycium ruthenicum* seedlings. CK, non-inoculated plants; NP, plants inoculated with *Neocamarosporium phragmitis*; AC, plants inoculated with *Alternaria chlamydospore*; MA, plants inoculated with *Microascus alveolaris*; WW, well-watered; DS, drought stress, respectively. Available N content of rhizosphere soil **(A)**, Available N content of rhizosphere soil **(B)**, Orangic matter content of rhizosphere soil **(C)**, and pH of rhizosphere soil **(D)**. The * symbol indicates the significant difference *P* < 0.05. The ** symbol indicates the significance level intervenes between *P* < 0.01. The *** symbol indicates difference is very significant, *P* < 0.001.

The results of two-way ANOVA showed that in addition to soil organic matter content, DSE and water had significant effects on soil available N, available P, and pH, respectively, while the interaction between DSE and water had significant effects on soil available P and pH (Table 4). There was no difference in soil available N among all treatments under WW, while soil available N with AC inoculation was lower than that in other treatments under DS (Figure 4A). Under WW, soil available P with DSE inoculation was lower than that of the control group, while soil available P with AC and MA inoculation was lower than that of the control group under DS (Figure 4B). Under WW, soil organic matter with AC inoculation was lower than that of other treatments, while there was no difference in soil organic matter for all treatments under DS (Figure 4C). Under WW, soil pH with NP inoculation was higher than that of other treatments, while soil pH with NP and AC inoculation was higher than that of the control group under DS (Figure 4D). The interaction effects of DSE and water on soil-available P and pH were obvious (Table 4).

**Table 4:** Two-way ANOVA of the effect of DSE and water condition on soil physicochemical parameters of *Lycium ruthenicum*.

	**DSE**		**Water**		DSE*Water	
	***F***	***P***	***F***	***P***	***F***	***P***
Available N (μg/g)	5.39	*	6.38	*	3.16	NS
Available P (μg/g)	8.60	**	11.26	***	6.03	*
Organic matter (mg/g)	3.99	NS	4.83	NS	0.58	NS
pH	5.13	*	7.20	**	5.26	*

The * symbol indicates the significant difference, P <0.05. The ** symbol indicates the significance level intervenes between P <0.01. The *** symbol indicates the difference is very significant at P <0.001.

### Soil Microbial Community Composition

**Figure 5:**
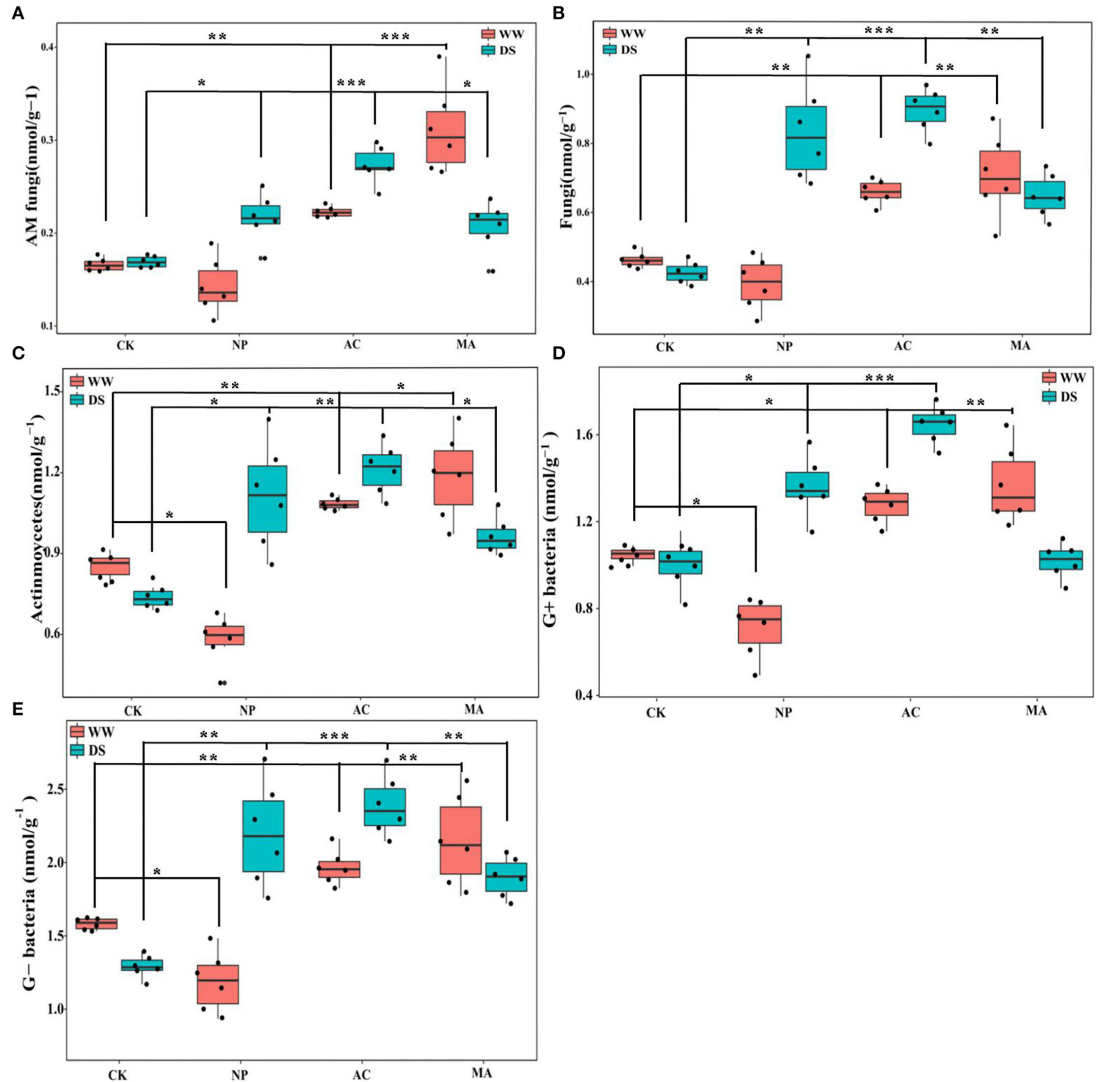
Effects of different dark septate endophyte (DSE) on soil microbial composition of *Lycium ruthenicum* seedlings. CK, non-inoculated plants; NP, plants inoculated with *Neocamarosporium phragmitis*; AC, plants inoculated with *Alternaria chlamydospore*; MA, plants inoculated with *Microascus alveolaris*; WW, well-watered; DS, drought stress, respectively. AM fungi content of rhizosphere soil **(A)**, Fungi content of rhizosphere soil **(B)**, Actinmoycetes content of rhizosphere soil **(C)**, G+ bacteria content of rhizosphere soil **(D)**, and Gbacteria content of rhizosphere soil **(E)**. The * symbol indicates the significant difference *P* < 0.05. The ** symbol indicates the significance level intervenes between *P* < 0.01. The *** symbol indicates difference is very significant, *P* < 0.001.

Water conditions were significantly correlated with soil microbial parameters. DSE significantly affected AM fungi and fungi, while the interaction between DSE and water significantly affected AM fungi and G+ bacteria (Table 5). AM fungi, soil fungi, actinomycetes, and G+ and G- bacteria levels with AC and MA inoculation were higher than that of the control group, while soil actinomycetes and G+ and G- bacteria levels with NP inoculation were lower than those in the control group under WW (Figure 5). The contents of AM fungi, soil fungi, actinomycetes, and G- bacteria with DSE inoculation were higher than those in the control group except for G+ bacteria under DS (Figure 5).

**Table 5:** Two-way ANOVA of the effect of DSE and water condition on soil microbial content of *Lycium ruthenicum*.

	**DSE**		**Water**		DSE*Water	
	***F***	***P***	***F***	***P***	***F***	***P***
AM fungi (nmol/g^−1^)	5.92	*	7.07	**	7.39	**
Fungi (nmol/g^−1^)	5.54	*	6.44	*	4.21	NS
Actinomycetes (nmol/g^−1^)	4.37	NS	6.92	*	3.06	NS
G+ bacteria (nmol/g^−1^)	6.28	NS	7.37	**	5.92	**
G– bacteria (nmol/g^−1^)	4.82	NS	5.73	*	4.38	NS

The * symbol indicates the significant difference P <0.05. The ** symbol indicates the significance level intervenes between P <0.01.

### Variation Partitioning Analysis

**Figure 6:**
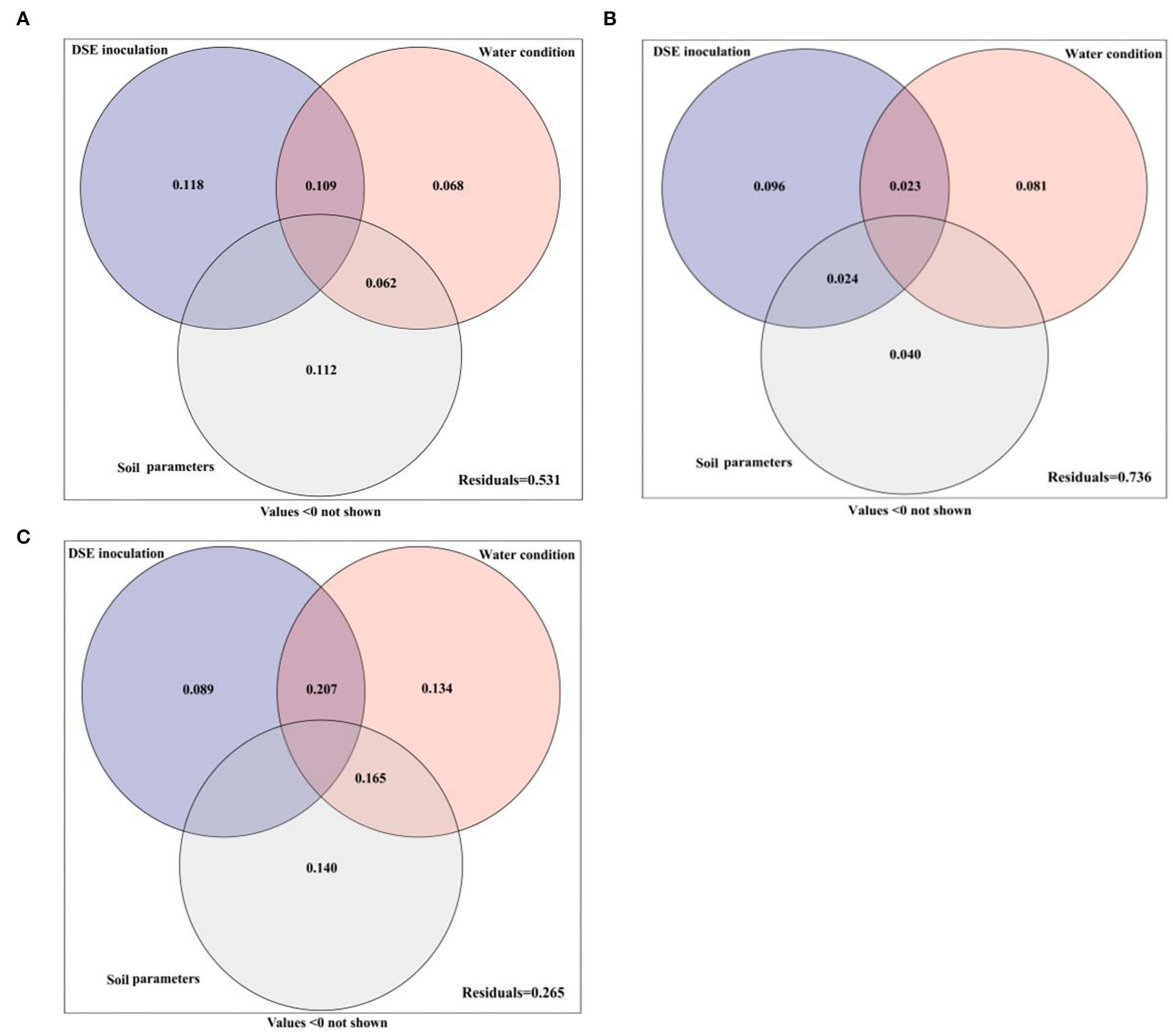
Variation partitioning of DSE, water condition, and soil parameter on plant morphological **(A)** and physiological **(B)** parameters and soil microbes **(C)**.

The variance partitioning analysis was performed to estimate the effects of DSE, water conditions, and soil parameters on the performance of host seedlings, and the contribution rates of different factors to the growth differences of host seedlings were quantitatively evaluated (Figure 6). The combined explanation of DSE, water conditions and soil parameters for plant morphology was 46.9%. The individual explanations were 11.8, 6.8, and 11.2%, respectively. The interaction between water and DSE accounted for 10.9%, while the interaction with soil parameters accounted for 6.2% (Figure 6A). DSE, water, and soil parameters together explained 26.4% of the total amount of the physiological parameters. The individual explanations were 9.6, 8.1, and 4.0%, respectively. The interaction between DSE and water was 2.3%, while that between DSE and soil parameters was 2.4% (Figure 6B). The comprehensive explanation of DSE, water, and soil parameters for soil microbes was 73.5%, and the individual explanations were 8.9, 13.4, and 14.0%, respectively. The interaction between water and DSE was 20.7%, while the interaction between water and soil parameters was 16.5% (Figure 6C).

### Correlation Analyses

**Figure 7:**
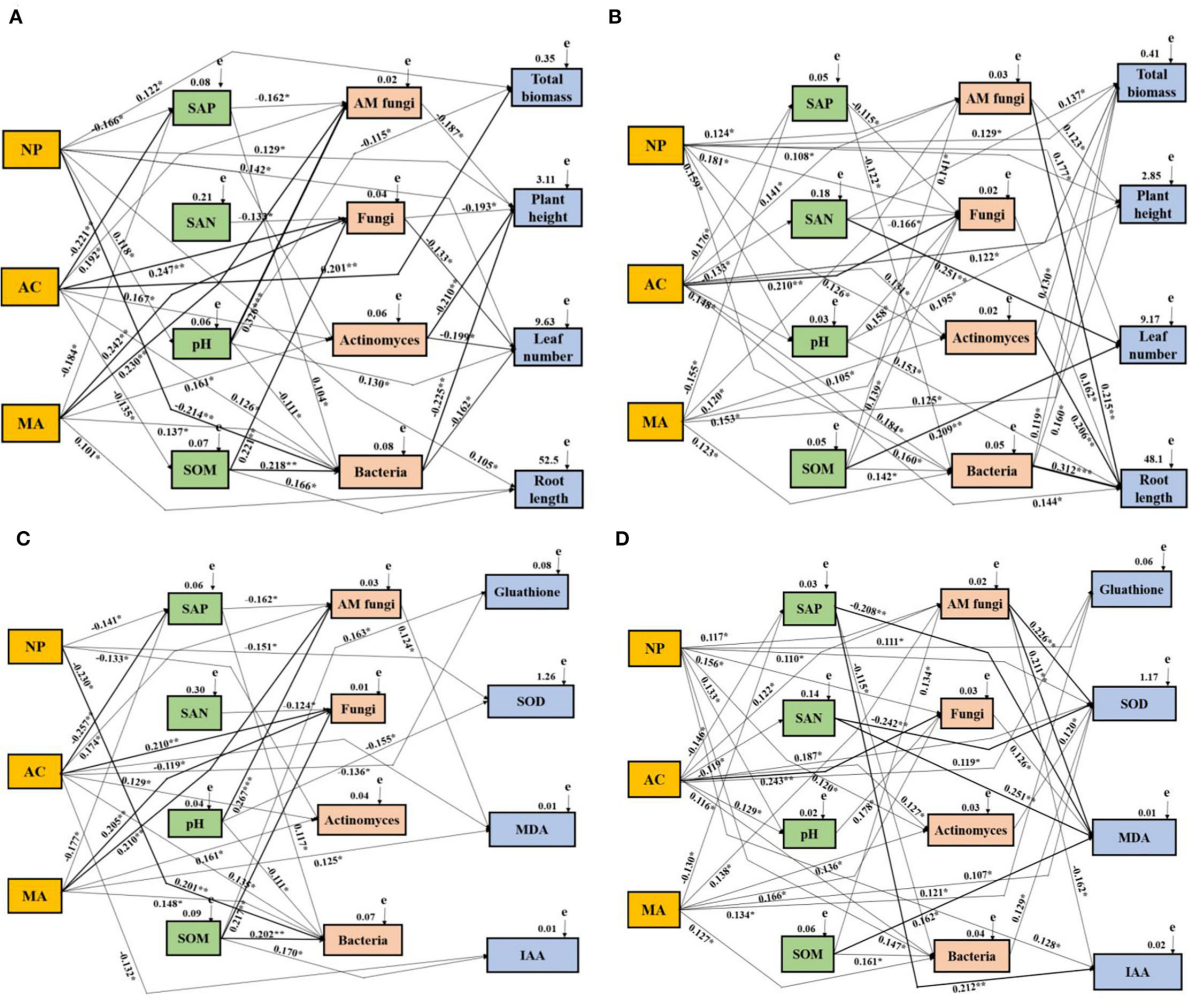
Structural equation model showing the causal relationships among DSE species, soil parameters, soil microbes, and growth and physiological parameters. The final model fitted the data well:maximum likelihood. Growth parameters under WW condition: **(A)** *X*^2^= 62.166, *df* = 9, *p* = 0.008, RMSEA (root mean square error of approximation) = 0.256, GFI (goodness-of-fit index) = 0.588, AIC (Akaike information criteria) = 168.167; Growth parameters under DS condition: **(B)** *X*^2^= 67.557, *df* =10, *p* = 0.006, RMSEA = 0.273, GFI = 0.569, AIC = 173.058; Physiological parameters under WW condition: **(C)** *X*^2^= 48.158, *df* = 6, *p* = 0.018, RMSEA = 0.225, GFI = 0.779, AIC = 152.037; Physiological parameters under DS condition: **(D)** *X*^2^= 55.666, *df* = 7, *p* = 0.011, RMSEA = 0.241, GFI = 0.633, AIC = 162.025. The solid and dashed lines indicate whether the path of action between different factors is significant or not, respectively. The width of the solid line indicates the strength of the effect between the different factors, and the numbers near the arrows indicate the normalized path coefficients (*p < 0.05, **p < 0.01, and ***p < 0.001). NP, *Neocamarosporium phragmitis*; AC, *Alternaria chlamydospore*; MA, *Microascus alveolaris*; SAP, soil available P; SAN, soil available N; SOM, soil organic matter.

The effects of DSE, water, soil properties, and soil microbes on the growth and physiological parameters of *L. ruthenicum* plants were studied by the Mantel test and the SEM. The Mantel test showed obvious relationships among DSE, water, growth parameter, physiological parameters, soil parameters, and microbes (Supplementary Tables 1–4). Based on the correlation coefficients (R-values), a SEM model was used to assess the relationship between DSE and all tested parameters under different water conditions. For the growth parameter under WW, NP positively influenced plant height, leaf number, root length, total biomass, and soil pH and negatively affected soil available P and bacteria. AC positively influenced total biomass, AM fungi, fungi, actinomyces, and bacteria, whereas it negatively affected soil available P and organic matter. MA positively influenced root length, AM fungi, fungi, bacteria, and actinomyces, and negatively affected soil available P. There was a positive correlation between soil available phosphorus and bacteria, but a negative correlation with AM fungi. Soil-available N negatively affected fungi. Soil pH positively influenced total biomass, and soil AM fungi and bacteria. Soil organic matter positively influenced GSH and IAA, soil fungi, and bacteria. AM fungi negatively affected plant height. Fungi, actinomyces, and bacteria negatively affected plant height and leaf number (Figure 7A). For growth parameters under DS, NP positively influenced plant height, leaf number and total biomass, soil pH, AM fungi, fungi, actinomyces, and bacteria. AC positively influenced total biomass, root length, soil pH, AM fungi, actinomyces, and bacteria. MA positively influenced total biomass, and soil AM fungi, fungi, actinomyces, and bacteria, whereas MA negatively affected soil available P. Soil available P positively influenced fungi and bacteria. There is a positive correlation between soil available N and total biomass, leaf number, and soil fungi. Soil pH positively influenced plant height, root length, and soil fungi. Soil organic matter had a positive affect on leaf number, growth of soil AM fungi, fungi and bacteria. AM fungi positively influenced plant height and root length. Fungi positively influenced root length. Actinomyces positively influenced total biomass and root length. Soil bacteria positively affect the total biomass, plant height, and root length (Figure 7B). For physiological parameters under WW, NP negatively affected SOD, soil available P, actinomyces, and bacteria. AC positively influenced soil AM fungi, fungi, actinomyces and bacteria, whereas AC negatively affected GSH, MDA, IAA, and soil available P. MA positively influenced MDA, and soil AM fungi, fungi, actinomyces, bacteria and available P. Soil available P positively influenced bacteria and AM fungi. Soil available N negatively affected soil fungi. There was a significant positive relationship between soil pH and SOD, AM fungi, and bacteria. Soil organic matter positively influenced GSH, IAA, and soil fungi and bacteria. AM fungi positively influenced MDA (Figure 7C). For physiological parameters under DS, NP positively influenced GSH, SOD, IAA, soil pH, AM fungi, fungi, actinomyces, and bacteria. AC positively influenced GSH, SOD, and soil pH, AM fungi, fungi, actinomyces, and bacteria, whereas AC negatively affected soil available P and available N. MA positively influenced SOD, AM fungi, fungi, actinomyces and bacteria and available P. Soil available P positively influenced IAA and MDA, and soil bacteria. Soil available N positively influenced MDA and SOD. Soil pH positively influenced fungi. Soil organic matter positively influenced MDA, and AM fungi and bacteria. AM fungi positively influenced SOD and MDA, whereas it negatively affected IAA. Fungi positively influenced MDA. Actinomyces and bacteria positively influenced SOD (Figure 7D).

## Discussion

Endophytic fungi can form a complex symbiotic relationship with plants, which may be neutral, reciprocal, or antagonistic due to environmental factors (Newsham, 2011; Surono and Narisawa, 2017). In the current study, inoculated DSE could successfully colonize the roots of *L. ruthenicum* and form typical hyphae and microsclerotia, while the response of the host plant to DSE colonization depended on DSE species, similar to previous studies (Newsham, 2011; Li et al., 2019). Inoculation with DSE is beneficial to plant growth and stress resistance, but different DSE strains have different abilities in promoting the host plant's performance (Mayerhofer et al., 2013). Zhang et al. (2012) had inoculated *P. chrysthemicola* and improved the biomass and root development of *L. ruthenicum*. Chu et al. (2019) had inoculated *P. chrysthemicola* and increased the biomass of *Pinus tabulaeformis* seedlings. Here, the results of two-way ANOVA found that DSE had a significant influence on the total biomass of *L. ruthenicum*. Compared with non-inoculated treatments, inoculated DSE enhanced the total biomass, plant height, and the number of spines, but decreased the root diameter. Based on the variance decomposition analysis, DSE had the greatest impact on the growth indicators of *L. ruthenicum*. The SEM model analysis revealed that only *N. phragmitis* had a direct effect on promoting the biomass, plant height, leaf number, and root length, while other DSE species indirectly promoted plant growth by influencing soil factors. Wilcox and Wang (1987) had found that some DSE strains were mild or severe pathogens when 4 DSE strains were inoculated into three trees, while others promoted the growth of the host plant. The current study found that the effects of DSE on plant performance under different water conditions depend on the fungal species involved, and fungal species may be a factor in the development of mutual symbiosis (Jumpponen, 2001).

Drought stress often affects plants and causes cell oxidative damage, while plants can adjust their antioxidant and osmotic systems under stress to protect plant tissues from damage (Bartels and Sunkar, 2005). It was found that GSH content, SOD activity, and soluble protein and proline content increased after inoculation with DSE under drought stress in the study. This indicates that DSE inoculation can effectively alleviate the adverse effects of water deficit on plants (Li and Yi, 2012). The results of the variance decomposition found that DSE species are also the main factor affecting the physiological activities of host plants, and the single explanation was 9.6%. The growth and development of plants and the ability to adapt to adversity are also inseparable from the participation of hormones. Research has shown that endophytic fungi can enhance the production of plant hormones and help host plants resist the adverse effects of abiotic stresses, thus, promoting plant growth and improving the growth environment of plants (Khan et al., 2012; Zhang et al., 2012). In this study, the IAA content of host plant roots was increased after inoculation with *N. phragmitis* under water stress compared to the control group. IAA is an important endogenous growth hormone in plants, and chlorophyll is an important photosynthetic pigment in plants. It has been reported that some DSE species can help host plants produce IAA and photosynthetic pigments (Waqas et al., 2012; Priyadharsini and Muthukumar, 2017). Inoculated *A. alternata* could increase the auxin content, and the growth and drought tolerance of wheat (Qiang et al., 2019). Therefore, we speculate that DSE may enhance the performance of *L. ruthenicum* by adjusting the IAA content in plant roots.

The soil environment is the substrate for plants to survive, and the difference in soil environment will directly influence plant growth and development and microbial community composition. Plants provide important habitats and nutrients for the growth of soil microbial communities (Zhang et al., 2016; Santos-Medellín et al., 2017), and the ecological functions of soil microorganisms will further influence plant growth and productivity. Plant morphology and physiology will change due to the interaction of different rhizosphere soil microorganisms (Dequiedt et al., 2011; Michiel et al., 2013). It has been found that DSE inoculation can improve soil properties (Casazza et al., 2017). DSE inoculation had significant influence on soil available N, available P, organic matter and pH, and DSE inoculation increased the uptake of soil available N and available P, which may be due to the mineralization of organic compounds and the release of fixed nutrients in the soil (Della Monica et al., 2015; Surono and Narisawa, 2017). Studies have shown that DSE hyphal network plays a key role in water and nutrient exchange between plants and soil under drought stress, which may be an effective measure for plants to resist adverse environments (Mandyam and Jumpponen, 2005; Peterson et al., 2008; Rodriguez et al., 2009). Soil microorganisms are particularly sensitive to environmental changes, and their functions and community composition respond quickly to changes in environmental conditions. This characteristic provides a reliable reference medium for the indirect study of plant root secretion and nutrient flow (Yang et al., 2013). There are many microbial communities in the roots of *L. ruthenicum* in the natural environment, and some soil microorganisms have the ability to adapt to adversity and promote plant performance (Pollastri et al., 2018). In this study, it was found that under normal water conditions, inoculation with *A. chlamydospore* and *M. alveolaris* increased the contents of AM fungi, fungi, bacteria, and actinomycetes in rhizosphere soil compared with the control group, while under drought stress, except for G+ bacteria, other microbial composition increased in all inoculation treatments. Variance decomposition and correlation showed that soil properties and water are the most important factors influencing soil microbial communities, reaching 14. 0% and 13. 4%, respectively. Under drought stress, AM fungi could positively influence plant height, root length, number of spines, and SOD and MDA content, and AM fungi were also negatively correlated with IAA. AM fungi can alleviate the drought of *Bombax ceiba* and *Saccharum arundinaceum*, improve their drought tolerance, and control tomato nematode disease (Marro et al., 2018). These results confirm that soil microorganisms can mediate plant resistance to drought through direct or indirect ways, which provides a basis to further clarify the mechanism of plant drought resistance and the cultivation of medicinal plants in dryland.

The current study found that all the tested DSE can effectively colonize the roots of *L. ruthenicum* and enhance plant growth and drought resistance through a comprehensive mechanism of improving plant morphological and physiological properties. These beneficial roles may be related to the changes in soil properties and microbial community of *L. ruthenicum* with DSE inoculation. Interestingly, all three DSE strains used in this experiment obviously promoted the contents of AM fungi under drought stress. *L. ruthenicum*, as an important medicinal and edible plant in the desert area, plays an important role in ameliorating desertification, while microorganisms inhabiting the rhizosphere of host plants form a mutualistic symbiosis, to help host plants better adapt to adverse environmental stresses. *N. phragmitis* can mitigate the negative effect of drought stress on host plants, especially having a beneficial influence on its growth and physiological index. Therefore, *N. phragmitis* has the potential to be used in the cultivation of *L. ruthenicum* in dryland.

## Data Availability Statement

The original contributions presented in the study are included in the article/Supplementary Materials, further inquiries can be directed to the corresponding author.

## Author Contributions

CH and XL conceived and designed the experiments and wrote the paper. CH and TH performed the experiments. CH, TH, and LT analyzed the data. All authors contributed to the article and approved the submitted version.

## Funding

This research was financially supported by the National Key R & D Program of China (2012ZX093604006).

## Conflict of Interest

The authors declare that the research was conducted in the absence of any commercial or financial relationships that could be construed as a potential conflict of interest.

## Publisher's Note

All claims expressed in this article are solely those of the authors and do not necessarily represent those of their affiliated organizations, or those of the publisher, the editors and the reviewers. Any product that may be evaluated in this article, or claim that may be made by its manufacturer, is not guaranteed or endorsed by the publisher.

## Supplementary Material

Supplementary Figure 1 Colonization and growth of dark septate endophytes (DSE) in the root system of *L. ruthenicum*. Hy, DSE hyphae; Mi, DSE microsclerotia. A, a: *Neocamarosporium phragmitis*; B, b: *Alternaria chlamydospore*; C. c: *Microascus alveolaris*.

Supplementary Table 1 Mantel tests showing correlationships (R values) between DSE species, soil physicochemical parameters, microbial community composition and plant growth indicators in WW conditions.

Supplementary Table 2 Mantel tests showing correlationships (R values) between DSE species, soil physicochemical parameters, microbial community composition and plant growth indicators under DS conditions.

Supplementary Table 3 Mantel tests showing correlationships (R values) between DSE species, soil physicochemical parameters, microbial community composition and plant physiological parameters under WW conditions.

Supplementary Table 4 Mantel tests showing correlationships (R values) between DSE species, soil physicochemical parameters, microbial community composition and plant physiological parameters under DS conditions.

## References

[B1] Addy H. D. Piercey M. M. Currah R. S. (2005). Microfungal endophytes in root. Can. J. Bot. 83, 1–13. 10.1139/b04-171

[B2] Albertsen A. Ravnskov S. Green H. Jensen D. F. Larsen J. (2006). Interactions between the external mycelium of the mycorrhizal fungus Glomus intraradices and other soil microorganisms as affected by organic matter. Soil Biol. Biochem. 38, 1008–1014. 10.1016/j.soilbio.2005.08.015

[B3] Anderson M. E (1985). Determination of glutathione and glutathione disulfide in biological samples. Method Enzymol. 113, 548–555. 10.1016/S0076-6879(85)13073-9 4088074

[B4] Azcón-Aguilar C. Palenzuela J. Roldán A. Bautist S. Vallejo R. Barea J. M. (2003). Analysis of the mycorrhizal potential in the rhizosphere of representative plant species from desertification- threatened Mediterranean shrublands. Agric. Ecosyst. Environ. Appl. Soil Ecol. 22, 29–37. 10.1016/S0929-1393(02)00107-5

[B5] Bai Y. Muller D. B. Srinivas G. Garrido-Oter R. Potthoff E. Rott M.. (2015). Functional overlap of the Arabidopsis leaf and root microbiota. Nature 528, 364–369. 10.1038/nature16192 26633631

[B6] Bartels D. Sunkar R. (2005). Drought and salt tolerance in plants. Crit. Rev. Plant Sci. 24, 23–58. 10.1080/07352680590910410

[B7] Bates L. S. Waldren R. P. Teare I. D. (1973). Rapid determination of free proline for water-stress studies. Plant Soil 39, 205–207. 10.1007/BF0001806020688380

[B8] Berthelot C. Perrin Y. Leyval C. Blaudez D. (2017). Melanization and ageing are not drawbacks for successful agro-transformation of dark septate endophytes. Fungal Biol. 121, 652–663. 10.1016/j.funbio.2017.04.004 28705394

[B9] Bever J. D. Morton J. B. Antonovics J. Schultz P. A. (1996). Host-dependent sporulation and species diversity of arbuscular mycorrhizal fungi in a mown grassland. J. Ecol. 84, 71–82. 10.2307/2261701

[B10] Bossio D. A. Scow K. M. (1998). Impacts of carbon and flooding on soil microbial communities: phospholipid fatty acid profiles and substrate utilization patterns. Microb. Ecol. 35, 265–278. 10.1007/s002489900082 9569284

[B11] Casazza G. Lumini E. Ercole E. Francesco D. Guerrina M. Arnulfo A.. (2017). The abundance and diversity of arbuscular mycorrhizal fungi are linked to the soil chemistry of screes and to slope in the Alpic paleo-endemic Berardia subacaulis. PLoS ONE 12, e0171866. 10.1371/journal.pone.0171866 28192471 PMC5305098

[B12] Cherif H. Marasco R. Rolli E. Ferjani R. Fusi M. Soussi A.. (2015). Oasis desert farming selects environment-specific date palm root endophytic communities and cultivable bacteria that promote resistance to drought. Environ. Microbiol. Rep. 7, 668–678. 10.1111/1758-2229.12304 26033617

[B13] Chu H. Wang C. Li Z. Wang H. H. Xiao Y. G. Chen J.. (2019). The dark septate endophytes and ectomycorrhizal fungi effect on *Pinus tabulaeformis* Carr. seedling growth and their potential effects to pine wilt disease resistance. Forests 10, 140. 10.3390/f10020140

[B14] Della Monica I. F. D. Saparrat M. C. N. Godeas A. M. Scervino J. M. (2015). The co-existence between DSE and AMF symbionts affects plant P pools through P mineralization and solubilization processes. Fungal Ecol. 17, 10–17. 10.1016/j.funeco.2015.04.004

[B15] Dequiedt S. Saby N. P. A. Lelievre M. Jolivet C. Thioulouse J. Toutain B.. (2011). Biogeographical patterns of soil molecular microbial biomass as influenced by soil characteristics and management. Global Ecol. Biogeogr. 20, 641–652. 10.1111/j.1466-8238.2010.00628.x

[B16] Goslee S. C. Urban D. L. (2007). The ecodist package for dissimilarity-based analysis of ecological data. J. Stat. Softw. 22, 1–19. 10.18637/jss.v022.i07

[B17] Han L. Shi J. He C. He X. (2021). Temporal and spatial dynamics of dark septate endophytes in the roots of Lycium ruthenicum in the desert region of Northwest China. Agronomy 11:648. 10.3390/agronomy11040648

[B18] Harrach B. D. Fodor J. Pogany M. Preuss J. Barna B. (2008). Antioxidant, ethylene and membrane leakage responses to powdery mildew infection of nearisogenic barley lines with various types of resistance. Eur. J. Plant Pathol. 121, 121–133. 10.1007/s10658-007-9236-3

[B19] He C. Wang W. Q. Hou J. L. (2019a). Characterization of dark septate endophytic fungi and improve the performance of licorice under organic residue treatment. Front. Microbiol. 10, 1364. 10.3389/fmicb.2019.01364 PMC6592127 31275282

[B20] He C. Wang W. Q. Hou J. L. (2019b). Plant growth and soil microbial impacts of enhancing licorice with inoculating dark septate endophytes under drought stress. Front. Microbiol. 10, 2277. 10.3389/fmicb.2019.02277 PMC6794389 31649632

[B21] Heiri O. Lotter A. F. Lemcke G. (2001). Loss on ignition as a method for estimating organic and carbonate content in sediments: reproducibility and comparability of results. J. Paleolimnol. 25, 101–110. 10.1023/A:1008119611481

[B22] Jumpponen A (2001). Dark septate endophytes - are they mycorrhizal? Mycorrhiza 11, 207–211. 10.1007/s005720100112

[B23] Jumpponen A. Trappe J. M. (1998). Dark septate endophytes: a review of facultative biotrophic root-colonizing fungi. New Phytol. 140, 295–310. 10.1046/j.1469-8137.1998.00265.x 33862835

[B24] Khan A. L. Hamayun M. Khan S. A. Kang S. M. Shinwari Z. K. Kamran M.. (2012). Pure culture of Metarhizium anisopliae LHL07 reprograms soybean to higher growth and mitigates salt stress. World J. Microbiol. Biotechnol. 28, 1483–1494. 10.1007/s11274-011-0950-9 22805930

[B25] Khan M. M. Islam E. Irem S. Akhtar K. Ashraf M. Y. Iqbal J.. (2018). Pb-induced phytotoxicity in para grass (*Brachiaria mutica*) and Castorbean (*Ricinus communis* L.): antioxidant and ultrastructural studies. Chemosphere 200, 257–265. 10.1016/j.chemosphere.2018.02.101 29494906

[B26] Kivlin S. N. Emery S. M. Rudgers J. A. (2013). Fungal symbionts alter plant responses to global change. Am. J. Bot. 100, 1445–1457. 10.3732/ajb.1200558 23757444

[B27] Li L. H. Yi H. L. (2012). Effect of sulfur dioxide on ROS production, gene expression and antioxidant enzyme activity in Arabidopsis plants. Plant Physiol. Biochem. 58, 46–53. 10.1016/j.plaphy.2012.06.009 22771435

[B28] Li X He C. He X. L. Su F. Hou L. F. Ren Y.. (2019). Dark septate endophytes improve the growth of host and non-host plants under drought stress through altered root development. Plant Soil 439, 259–272. 10.1007/s11104-019-04057-2

[B29] Li X. He X. L. Hou L. F. Ren Y. Wang S. J. Su F. (2018). Dark septate endophytes isolated from a xerophyte plant promote the growth of *Ammopiptanthus mongolicus* under drought condition. Sci. Rep. 8, 7896–7906. 10.1038/s41598-018-26183-0 29785041 PMC5962579

[B30] Lu L.H Zou Y. N. Wu Q. S. (2019). Mycorrhizas mitigate soil replant disease of peach through regulating root exudates, soil microbial population, and soil aggregate stability. Commun. Soil Sci. Plan. 50, 909–921. 10.1080/00103624.2019.1594882

[B31] Mandyam K. Jumpponen A. (2005). Seeking the elusive function of the root-colonising dark septate endophytic fungi. Stud. Mycol. 53, 173–189. 10.3114/sim.53.1.173

[B32] Mandyam K. Loughin T. Jumpponen A. (2010). Isolation and morphological and metabolic characterization of common endophytes in annually burned tallgrass prairie. Mycologia 102, 813–821. 10.3852/09-212 20648749

[B33] Marro N. Caccia M. Doucet M. E. Cabello M. Becerra A. Lax P. (2018). Mycorrhizas reduce tomato root penetration by false root-knot nematode *Nacobbus aberrans*. Agric. Ecosyst. Environ. Appl. Soil Ecol. 124, 262–265. 10.1016/j.apsoil.2017.11.011

[B34] Mayerhofer M. S. Kernaghan G. Harper K. A. (2013). The effects of fungal root endophytes on plant growth: a meta-analysis. Mycorrhiza 23, 119–128. 10.1007/s00572-012-0456-9 22983627

[B35] Michiel V. Wolf A. B. Jennings S. J. Kowalchuk G. A. (2013). Micro-scale determinants of bacterial diversity in soil. FEMS Microbiol. Rev. 37, 936–954. 10.1111/1574-6976.12023 23550883

[B36] Muthukumar T. Sathya R. (2017). Endorhizal fungal association and colonization patterns in Solanaceae. Polish. Bot. J. 62, 287–299. 10.1515/pbj-2017-0016

[B37] Newsham K. K (2011). A meta-analysis of plant responses to dark septate root endophytes. New Phytol. 190, 783–793. 10.1111/j.1469-8137.2010.03611.x 21244432

[B38] Peever T. L. Higgins V. J. (1989). Electrolyte leakage, lipoxygenase, and lipid peroxidation induced in tomato leaf tissue by specific and nonspecific elicitors from Cladosporium fulvum. Plant Physiol. 90, 867–875. 10.1104/pp.90.3.867 16666890 PMC1061813

[B39] Peterson R. L. Wagg C. Pautler M. (2008). Associations between microfungal endophytes and roots: do structural features indicate function? Botany 86, 445–456. 10.1139/B08-016

[B40] Pollastri S. Savvides A. Pesando M. Lumini E. Volpe M. G. Ozudogru E. A.. (2018). Impact of two arbuscular mycorrhizal fungi on *Arundo donax* L. response to salt stress. Planta 247, 573–585. 10.1007/s00425-017-2808-3 29124326

[B41] Priyadharsini P. Muthukumar T. (2017). The root endophytic fungus Curvularia geniculata from Parthenium hysterophorus roots improves plant growth through phosphate solubilization and phytohormone production. Fungal Ecol. 27, 69–77. 10.1016/j.funeco.2017.02.007

[B42] Qiang X. J. Ding J. J. Lin W. Li Q. Xu C. Zheng Q.. (2019). Alleviation of the detrimental effect of water defcit on wheat (*Triticum aestivum* L.) growth by an indole acetic acid-producing endophytic fungus. Plant Soil 439, 373–391. 10.1007/s11104-019-04028-7

[B43] Rodriguez R. J. White J. F. Arnold A. E. Redman R. S. (2009). Fungal endophytes: diversity and functional roles. New Phytol. 182, 314–330. 10.1111/j.1469-8137.2009.02773.x 19236579

[B44] Saleem M. Asghar H. N. Zahir Z. A. Shahid. (2018). Impact of lead tolerant plant growth promoting rhizobacteria on growth, physiology, antioxidant activities, yield and lead content in sunflower in lead contaminated soil. Chemosphere 195, 606–614. 10.1016/j.chemosphere.2017.12.117 29278850

[B45] Santos-Medellín C. Edwards J. Liechty Z. Nguyen B. Sundaresan V. (2017). Drought stress results in a compartment-specific restructuring of the rice root-associated microbiomes. MBio 8, e00764–e00717. 10.1128/mBio.00764-17 28720730 PMC5516253

[B46] Sharma P. Jha A. B. Dubey R. S. Mohammad P. (2012). Reactive oxygen species, oxidative damage, and antioxidative defense mechanism in plants under stressful conditions. J. Bot. 2012, 1–26. 10.1155/2012/21703727269705

[B47] Shi Z. Mickan B. Feng G. Chen Y. L. (2015). Arbuscular mycorrhizal fungi improved plant growth and nutrient acquisition of desert ephemeral Plantago minuta under variable soil water conditions. J. Arid. Land 7, 414–420. 10.1007/s40333-014-0046-0

[B48] Surono and Narisawa, K (2017). The dark septate endophytic fungus Phialocephala fortinii, is a potential decomposer of soil organic compounds and a promoter of Asparagus officinalis growth. Fungal Ecol. 28, 1–10. 10.1016/j.funeco.2017.04.001

[B49] Trabelsi D. Mhamdi R. (2013). Microbial inoculants and their impact on soil microbial communities: a review. Biomed Res. Int. 2013, 863240. 10.1155/2013/863240 PMC3728534 23957006

[B50] Waqas M. Khan A. L. Kamran M. Hamayun M. Kang S. M. Kim Y. H.. (2012). Endophytic fungi produce gibberellins and indoleacetic acid and promotes host-plant growth during stress. Molecules 17, 10754–10773. 10.3390/molecules170910754 22960869 PMC6268353

[B51] Wilcox H. E. Wang C. J. K. (1987). Ectomycorrhizal and ectendomycorrhizal associations of Phialophorafnlandia with Pinusresinosa, Picearubens, and Betula alleghaniensis. Can. J. Forest Res. 17, 976–990. 10.1139/x87-152

[B52] Wu L. Q. Lv Y. L. Meng Z. X. Chen J. Guo S. X. (2010). The promoting role of an isolate of dark-septate fungus on its host plant Saussurea involucrata Kar. et Kir. Mycorrhiza 20, 127–135. 10.1007/s00572-009-0268-8 19707800

[B53] Yakti W. Kovács G. M. Vági P. Franken P. (2018). Impact of dark septate endophytes on tomato growth and nutrient uptake. Plant Ecol. Diver. 11, 637–648. 10.1080/17550874.2019.1610912

[B54] Yang J. W. Zhang N. Ma C. Y. Qu Y. Si H. J. Wang D. (2013). Prediction and verification of micro RNAs related to proline accumulation under drought stress in potato. Comput. Biol. Chem. 46, 48–54. 10.1016/j.compbiolchem.2013.04.006 23764530

[B55] Zhang C. Liu F. Kong W. W. He Y. (2015). Application of visible and near-infrared hyperspectral imaging to determine soluble protein content in oilseed rape leaves. Sensors 15, 16576–16588. 10.3390/s150716576 26184198 PMC4541894

[B56] Zhang H. H. Tang M. Chen H.. (2012). Effects of a dark-septate endophytic isolate LBF-2 on the medicinal plant *Lycium barbarum* L. J. Microbiol. 50, 91–96. 10.1007/s12275-012-1159-9 22367942

[B57] Zhang S. Gan Y. Xu B. (2016). Application of plant-growth-promoting fungi Trichoderma longibrachiatum T6 enhances tolerance of wheat to salt stress through improvement of antioxidative defense system and gene expression. Front. Plant Sci. 7, 1405. 10.3389/fpls.2016.01405 PMC5023664 27695475

[B58] Zhao D. K. Li T. Shen M. Wang J. L. Zhao Z. W. (2015). Diverse strategies conferring extreme cadmium (Cd) tolerance in the dark septate endophyte (DSE), Exophiala pisciphila: evidence from RNA-seq data. Microbiol. Res. 170, 27–35. 10.1016/j.micres.2014.09.005 25294257

[B59] Zheng G. Q. Zheng Z. Y. Xu X. Hu Z. H. (2010). Variation in fruit sugar composition of *Lycium barbarum* L. and *Lycium chinense* Mill. of different regions and varieties. Biochem. Syst. Ecol. 38, 275–284. 10.1016/j.bse.2010.01.008

[B60] Zuo Y. L. Su F. He X. L. Li M. (2020). Colonization by dark septate endophytes improves the growth of Hedysarum scoparium under multiple inoculum levels. Symbiosis 82, 201–214. 10.1007/s13199-020-00713-9

