# Supplementary material for: Effects of Dark Septate Endophytes on the Performance and Soil Microbia of Lycium ruthenicum Under Drought Stress
Source: Front Plant Sci. 2022 Jun 2;13:898378. doi: 10.3389/fpls.2022.898378 (PMC9201775; doi:10.3389/fpls.2022.898378)
Supplement: Supplementary Figure 1 — Colonization and growth of dark septate endophytes (DSE) in the root system of L. ruthenicum. Hy, DSE hyphae; Mi, DSE microsclerotia. A, a: Neocamarosporium phragmitis; B, b: Alternaria chlamydospore; C. c: Microascus alveolaris. [file Data_Sheet_1.doc]

**Fig.S1**

Table S1 Mantel tests showing correlationships (R values) between DSE species, soil physicochemical parameters, microbial community composition and plant growth indicators in WW conditions.

| Variable | NP | AC | MA | SAP | SAN | pH | SOM | AM | Fungi | Act | Bacteria | TB | PLH | LN | RL |
| --- | --- | --- | --- | --- | --- | --- | --- | --- | --- | --- | --- | --- | --- | --- | --- |
| NP | 1 |  |  |  |  |  |  |  |  |  |  |  |  |  |  |
| AC | 0.07 | 1 |  |  |  |  |  |  |  |  |  |  |  |  |  |
| MA | 0.05 | -0.07 | 1 |  |  |  |  |  |  |  |  |  |  |  |  |
| SAP | -0.11* | -0.21** | -0.16* | 1 |  |  |  |  |  |  |  |  |  |  |  |
| SAN | -0.03 | -0.05 | 0.03 | 0.07 | 1 |  |  |  |  |  |  |  |  |  |  |
| pH | 0.13* | 0.01 | 0.02 | 0.02 | -0.06 | 1 |  |  |  |  |  |  |  |  |  |
| SOM | 0.06 | 0.14* | 0.05 | -0.11* | 0.08 | -0.14* | 1 |  |  |  |  |  |  |  |  |
| AM | -0.06 | 0.17* | 0.23** | -0.14* | 0.03 | 0.33*** | 0.06 | 1 |  |  |  |  |  |  |  |
| Fungi | -0.04 | 0.26** | 0.26** | -0.05 | -0.15* | 0.06 | 0.23** | 0.02 | 1 |  |  |  |  |  |  |
| Act | -0.16* | 0.14* | 0.18* | 0.02 | -0.06 | -0.02 | 0.09 | -0.06 | -0.03 | 1 |  |  |  |  |  |
| Bacteria | 0.13* | 0.11* | 0.13* | 0.12* | 0.05 | 0.18* | 0.26** | -0.12* | -0.08 | 0.06 | 1 |  |  |  |  |
| TB | 0.18* | 0.19* | 0.04 | -0.03 | -0.04 | -0.12* | -0.07 | -0.02 | 0.02 | 0.01 | 0.05 | 1 |  |  |  |
| PLH | 0.15* | 0.06 | 0.06 | 0.08 | 0.05 | -0.06 | -0.04 | -0.15* | -0.17* | -0.23** | 0.25** | 0.12* | 1 |  |  |
| LN | 0.13* | -0.02 | 0.01 | 0.01 | 0.09 | 0.15* | 0.05 | 0.01 | -0.11* | -0.20** | 0.19* | 0.09 | 0.11* | 1 |  |
| RL | 0.11* | 0.04 | 0.12* | 0.09 | 0.07 | -0.09 | 0.18* | 0.03 | 0.07 | -0.06 | -0.05 | 0.27** | 0.08 | -0.04 | 1 |

Table S2 Mantel tests showing correlationships (R values) between DSE species, soil physicochemical parameters, microbial community composition and plant growth indicators under DS conditions.

| Variable | NP | AC | MA | SAP | SAN | pH | SOM | AM | Fungi | Act | Bacteria | TB | PLH | LN | RL |
| --- | --- | --- | --- | --- | --- | --- | --- | --- | --- | --- | --- | --- | --- | --- | --- |
| NP | 1 |  |  |  |  |  |  |  |  |  |  |  |  |  |  |
| AC | 0.04 | 1 |  |  |  |  |  |  |  |  |  |  |  |  |  |
| MA | 0.08 | -0.05 | 1 |  |  |  |  |  |  |  |  |  |  |  |  |
| SAP | -0.02 | -0.17* | -0.13* | 1 |  |  |  |  |  |  |  |  |  |  |  |
| SAN | 0.01 | -0.13* | 0.04 | 0.03 | 1 |  |  |  |  |  |  |  |  |  |  |
| pH | 0.17* | 0.15* | -0.03 | -0.07 | -0.02 | 1 |  |  |  |  |  |  |  |  |  |
| SOM | 0.08 | 0.02 | 0.06 | -0.06 | 0.06 | -0.17* | 1 |  |  |  |  |  |  |  |  |
| AM | 0.16* | 0.12* | 0.16* | -0.04 | 0.09 | 0.09 | 0.14* | 1 |  |  |  |  |  |  |  |
| Fungi | 0.21** | 0.19* | 0.14* | -0.10* | -0.17* | 0.15* | 0.12* | 0.06 | 1 |  |  |  |  |  |  |
| Act | 0.14* | 0.14* | 0.10* | 0.05 | 0.02 | 0.04 | 0.05 | -0.15* | -0.08 | 1 |  |  |  |  |  |
| Bacteria | 0.16* | 0.12* | 0.13* | -0.15* | 0.03 | 0.11* | 0.16* | -0.08 | -0.13** | 0.09 | 1 |  |  |  |  |
| TB | 0.13* | 0.14* | 0.11* | 0.07 | 0.15* | 0.08 | -0.06 | -0.03 | 0.06 | 0.15* | 0.13* | 1 |  |  |  |
| PLH | 0.15* | 0.05 | 0.02 | -0.02 | 0.02 | 0.22** | 0.05 | -0.13* | 0.09 | 0.06 | 0.18* | 0.21** | 1 |  |  |
| LN | 0.19* | 0.02 | 0.04 | 0.03 | 0.23** | 0.07 | 0.22** | 0.01 | 0.02 | -0.04 | 0.03 | 0.12* | 0.17* | 1 |  |
| RL | 0.03 | 0.16* | 0.02 | -0.05 | 0.01 | 0.18* | -0.04 | 0.24** | 0.15* | 0.02 | 0.35*** | 0.15* | 0.14* | 0.07 | 1 |

Table S3 Mantel tests showing correlationships (R values) between DSE species, soil physicochemical parameters, microbial community composition and plant physiological parameters under WW conditions.

| Variable | NP | AC | MA | SAP | SAN | pH | SOM | AM | Fungi | Act | Bacteria | Glu | SOD | MDA | GH |
| --- | --- | --- | --- | --- | --- | --- | --- | --- | --- | --- | --- | --- | --- | --- | --- |
| NP | 1 |  |  |  |  |  |  |  |  |  |  |  |  |  |  |
| AC | 0.04 | 1 |  |  |  |  |  |  |  |  |  |  |  |  |  |
| MA | 0.07 | -0.04 | 1 |  |  |  |  |  |  |  |  |  |  |  |  |
| SAP | -0.14* | -0.26** | -0.19* | 1 |  |  |  |  |  |  |  |  |  |  |  |
| SAN | -0.05 | -0.02 | 0.05 | 0.04 | 1 |  |  |  |  |  |  |  |  |  |  |
| pH | 0.09 | 0.03 | 0.03 | 0.01 | -0.07 | 1 |  |  |  |  |  |  |  |  |  |
| SOM | 0.03 | 0.08 | 0.07 | -0.07 | 0.06 | -0.11* | 1 |  |  |  |  |  |  |  |  |
| AM | 0.05 | 0.15* | 0.18* | -0.17* | 0.02 | 0.26** | 0.08 | 1 |  |  |  |  |  |  |  |
| Fungi | -0.03 | 0.22** | 0.22** | -0.08 | -0.09 | 0.05 | 0.25** | 0.02 | 1 |  |  |  |  |  |  |
| Act | -0.14* | 0.13* | 0.17* | 0.02 | -0.04 | -0.04 | 0.06 | -0.06 | 0.02 | 1 |  |  |  |  |  |
| Bacteria | 0.17* | 0.15* | 0.11* | 0.12* | 0.06 | 0.13* | 0.18* | -0.12* | -0.11* | 0.04 | 1 |  |  |  |  |
| Glu | 0.03 | -0.12* | 0.07 | 0.02 | 0.04 | 0.07 | 0.15* | -0.02 | 0.05 | 0.03 | 0.04 | 1 |  |  |  |
| SOD | -0.19* | 0.01 | 0.01 | 0.09 | -0.03 | -0.14* | 0.01 | -0.15* | 0.03 | 0.05 | 0.06 | -0.01 | 1 |  |  |
| MDA | 0.05 | -0.15* | 0.04 | 0.01 | 0.01 | 0.05 | 0.02 | 0.01 | 0.08 | 0.04 | 0.08 | 0.04 | 0.09 | 1 |  |
| IAA | 0.02 | -0.13* | 0.02 | 0.04 | 0.05 | 0.06 | 0.16* | 0.03 | -0.04 | -0.03 | -0.06 | 0.06 | 0.04 | 0.03 | 1 |

Table S4 Mantel tests showing correlationships (R values) between DSE species, soil physicochemical parameters, microbial community composition and plant physiological parameters under DS conditions.

| Variable | NP | AC | MA | SAP | SAN | pH | SOM | AM | Fungi | Act | Bacteria | Glu | SOD | MDA | GH |
| --- | --- | --- | --- | --- | --- | --- | --- | --- | --- | --- | --- | --- | --- | --- | --- |
| NP | 1 |  |  |  |  |  |  |  |  |  |  |  |  |  |  |
| AC | 0.02 | 1 |  |  |  |  |  |  |  |  |  |  |  |  |  |
| MA | 0.04 | -0.08 | 1 |  |  |  |  |  |  |  |  |  |  |  |  |
| SAP | 0.01 | -0.16* | -0.12* | 1 |  |  |  |  |  |  |  |  |  |  |  |
| SAN | 0.03 | -0.10* | 0.04 | 0.01 | 1 |  |  |  |  |  |  |  |  |  |  |
| pH | 0.13* | 0.12* | -0.06 | 0.03 | -0.05 | 1 |  |  |  |  |  |  |  |  |  |
| SOM | 0.07 | 0.04 | 0.03 | -0.11* | 0.03 | -0.13* | 1 |  |  |  |  |  |  |  |  |
| AM | 0.16* | 0.12* | 0.14* | -0.06 | 0.08 | 0.07 | 0.10* | 1 |  |  |  |  |  |  |  |
| Fungi | 0.14* | 0.20** | 0.17* | -0.04 | -0.06 | 0.13* | 0.17** | 0.09 | 1 |  |  |  |  |  |  |
| Act | 0.11* | 0.15* | 0.13* | 0.02 | 0.01 | 0.02 | 0.08 | -0.10* | -0.06 | 1 |  |  |  |  |  |
| Bacteria | 0.17* | 0.15* | 0.09 | 0.14* | 0.05 | 0.08 | 0.14* | -0.05 | -0.16* | 0.07 | 1 |  |  |  |  |
| Glu | 0.13* | 0.11* | 0.14* | -0.03 | 0.01 | -0.05 | 0.02 | -0.02 | -0.03 | -0.02 | 0.02 | 1 |  |  |  |
| SOD | 0.19* | 0.01 | 0.10* | -0.08 | -0.24** | 0.01 | 0.03 | 0.21** | 0.05 | 0.11* | 0.12* | 0.03 | 1 |  |  |
| MDA | 0.01 | 0.03 | 0.02 | -0.17* | 0.22** | 0.03 | 0.02 | 0.18* | 0.15* | 0.09 | 0.05 | 0.02 | 0.12* | 1 |  |
| IAA | 0.15* | 0.02 | 0.03 | 0.26** | 0.07 | 0.02 | 0.05 | -0.13* | 0.04 | 0.05 | 0.04 | 0.09 | 0.06 | 0.01 | 1 |
